# Cultural entrainment of motor skill development: Learning to write *hiragana* in Japanese primary school

**DOI:** 10.1002/dev.21536

**Published:** 2017-06-13

**Authors:** Tetsushi Nonaka

**Affiliations:** ^1^ Graduate School of Human Development and Environment Kobe University Japan

**Keywords:** differentiation, handwriting, learning, motor skill development, tool use

## Abstract

The aim of the present study was to examine how the social norms shared in a classroom environment influence the development of movement dynamics of handwriting of children who participate in the environment. To look into this issue, the following aspects of the entire period of classroom learning of *hiragana* letters in Japanese 1st graders who had just entered primary school were studied: First, the structure of classroom events and the specific types of interaction and learning within such environment were described. Second, in the experiment involving 6‐year‐old children who participated in the class, writing movements of children and their changes over the period of *hiragana* education were analyzed for each stroke composing letters. It was found that writing movement of children became differentiated in a manner specific to the different types of stroke endings, to which children were systematically encouraged to attend in the classroom. The results provide a detailed description of the process of how dynamics of fine motor movement of children is modulated by the social norms of a populated, classroom environment in a non‐Latin alphabet writing system.

## 1 INTRODUCTION

Handwriting is a “frozen gesture,” the persistent traces of manual movements. The path of the progressive movement of the tip of the graphic tool held in hand alters the light‐reflecting capacity of a surface permanently in such a way to specify graphic symbols. The tracings afford viewing to others because they structure the optic array (Gibson & Yonas, 1967). Hence, it is an act of communication, a social act. Writing is generally assumed to be one of the hallmarks of our species (Leroi‐Gourhan, 1993). Yet as we all know, human babies are not born writing. Rather, this instrumentally assisted, inscriptive gesture is an acquired skill that develops slowly in an environment that includes caregivers and a range of supporting objects. In addition, unlike skills such as reaching, pointing, walking, or speech, the skill of handwriting does not just develop even if a child is reared in the normal human environment, but usually requires extensive social training (Vygotsky, 1978c). Children are known to spend much of their school day performing handwriting (McHale & Cermak, 1992). Despite the use of computers, handwriting remains an important developmental skill for a child to master (Feder & Majnemer, 2007).

Handwriting requires the control of continuous hand movements in such a way to leave a desired trajectory of the tip of the pen on a two‐dimensional writing surface. The task, therefore, has traditionally been viewed as a task of controlling fine motor movements, different aspects of which have been investigated by a number of researchers (van Gemmert & Contreras‐Vidal, 2015). For example, Greer and Lockman (1998) asked preschoolers to draw horizontal and vertical lines with pens at different locations on a page. These researchers found a reduction in variability both in grip pattern and pen‐surface positioning between 3 and 5 years of age, and suggested that such developmental changes in the variability reflect the process of discovering forms of writing that maximize stability in orienting a tool to a surface such that certain spatial relations are maintained (Greer & Lockman, 1998). Newell and van Emmerik (1989) investigated the development of motor coordination in handwriting not in children but in adults, who practiced writing with their non‐dominant hand their signature and cursive e's 1,000 and 10,000 times, respectively. The effect of practice turned out very small, implying that the acquisition of the skill may take much more practice than has been applied in experimental studies. In addition, it was found that in right‐handers, the non‐dominant limb links were more tightly frozen than was the case with the dominant limb, while this was not a case in left‐handers, suggesting the influence of writing direction on the acquisition of coordination patterns (Newell & van Emmerik, 1989). More recently, Duval and co‐workers (Duval, Rémi, Plamondon, Vaillant, & O'Reilly, 2015) studied the graphomotor skills of kindergarten children who produced pre‐calligraphic trajectories, using different indexes of rapidity, fluidity, and regularity of their pen tip movements.

One of the curious facts about the previous studies on the development of handwriting is that the tasks performed by participants were often not the actual task of writing meaningful letters, but that of drawing or tracing of nonsense graphic forms such as straight lines and ellipses (Danna, Enderli, Athènes, & Zanone, 2012; Greer & Lockman, 1998), continuous lines of loop patterns (Bosga‐Stork, Bosga, & Meulenbroek, 2011), or other abstract letter‐like patterns (Duval et al., 2015; Jongbloed‐Pereboom, Peeters, Overvelde, Nijhuis‐van der Sanden, & Steenbergen, 2015). In these studies, the task of handwriting was viewed as the task of forming and producing a desired spatial trajectory of the tip of a tool held in hand. The development of the skill to plan and generate fine movements to precisely control the endpoint of a tool is, no doubt, an important topic of research. However, the research on the skills of tracing meaningless graphic forms inevitably leaves out an account of what it is that makes one's handwriting “meaningful,” affording discrimination by others in a populated environment. And one is left to wonder whether it is possible to separate learning to control bodily movements involved in writing from learning to write in a socially meaningful manner.

In my view, the developmental research on handwriting needs to be complemented by something like the Vygotskian perspective (Vygotsky, 1978a) and the Gibsonian perspective (Gibson, E. J., 1971; Gibson J. J., 2015). Despite their different emphases, both Vygotsky and the Gibson's went beyond the physical/social dichotomy in important ways by placing primacy on the terrestrial, populated environment in which the development of human skills literally takes place. Vygotsky emphasized, among other things, the socially organized, future‐oriented, yet nonlinear nature of child development (Vygotsky, 1978b). Human learning is prospective in a sense that it aims, in advance, for a new stage of the developmental process with the guidance of adults. Thereby, children grow into the life of those around them (Vygotsky, 1978b). Vygotsky, who viewed the development of writing as an interesting paradigm for the problem of the relation between learning and development, referred to the skill as “a complex cultural activity (Vygotsky, 1978c, p.118)” which develops along the path that it does because of cultural entrainment towards functioning in a populated environment.

E. J. Gibson extensively studied how children learn to read (Gibson, 1965, 1970, 1971, 1975; Gibson & Levin, 1975; Gibson, Gibson, Pick, & Osser, 1962). Through a series of experiments, Gibson showed that the ontogeny of reading in children involves progressive differentiation of the “distinctive features” of graphic information available in a given writing system (Gibson, 1971). Distinctive features are relational, not absolute like building blocks (Gibson, 1975). For example, 4‐year olds may confuse Latin alphabet letters i, j, and t. Yet, adults may focus their attention to distinctive topological features such as how the two lines are laid out relative to one another, or curve‐straight contrast at the end of a line to discriminate one letter from another, even if the letters are poorly handwritten and far from being similar or congruent to printed fonts. In other words, distinctive features are what remain invariant over a number of transformations that are irrelevant or noncritical for differentiating the symbols (Gibson, 1975). Likewise, in the act of writing, some relational features of traces left by the movement are expected to be critical and more tightly controlled than others, because these distinctive features must be made available in order for lines on paper to afford discrimination by other members of a language community. This view is in line with the argument presented by Bernstein from the perspective of motor control: “the human motor system cannot attain any high degree of metric proficiency, but it can be said that our motor system is very sensitive to topological distinctions of higher orders … . It is sufficient, for example, to draw attention to handwriting … that the letter *A* belongs to a single topological class of the first order no matter how or by whom it is written .… the analysis of which is not yet practicable for us because of our lack of acquaintance at present with whatever may constitute higher topological orders and what properties we must ascribe to them (Bernstein, 1984, pp.105–106).” If this line of reasoning is correct, then “what is learned” by children acquiring the skill of handwriting may not be so much the formation and reproduction of a graphic trajectory, but the ways to differentiate meaningful topological variations of lines by the movement of a writing implements on a writing surface. Arguably, addressing this point requires us to begin to take seriously the contexts of values in the populated environment to which the development of the skill progressively adapts (c.f., Bril, 1986; Gibson, 1950; Mauss, 1973; Tomasello, 1999; Vygotsky, 1978a).

What is important, but has not been studied, is how the populated environment entrains the perceiving and acting involved in handwriting skills of children. To explore this issue, the present study investigates the handwriting skill development of children in relation to the classroom environment in the beginning of handwriting education in Japanese primary school. By considering a syllabary that is unfamiliar to Latin alphabet communities, I aim to highlight and emphasize how the process of development of motor skills in handwriting is related to those dimensions that are critical in the populated environment where learning occurs.

**Figure 1:**
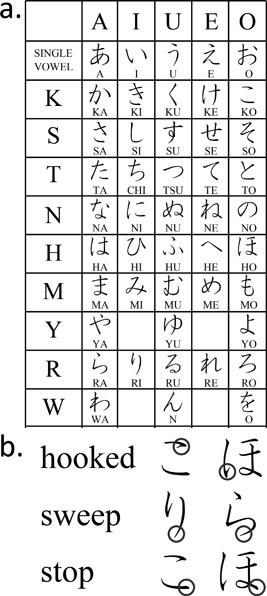
a. A Japanese syllabary *hiragana*. b. Three ways to end a stroke: “hooked,” “sweep,” and “stop”

The present study will concern a Japanese syllabary called *hiragana*. To add to the borrowed Chinese logograms, the Japanese developed two syllabaries: *hiragana* and *katakana*, each of which has 46 letters (Figure 1a). It is possible to write Japanese using only either one of the syllabaries. But in practice, Japanese books are written with a combination of Chinese characters and the syllabaries, which makes them much easier to read than the syllabary alone because the Chinese characters serve to provide clues for dividing the text into word or meaning units (Sakamoto & Makita, 1973). Japanese prints texts from top‐to‐bottom from right‐to‐left, although both these vertical arrangements and the other horizontal, left‐to‐right printing are commonly used today. Each *hiragana* letter, as well as each Chinese character, is written according to a set sequence of strokes. To learn how to write a letter is to learn how to trace each of its elements in a precise order, which is itself determined by a certain number of rules. Among others, there are following three types of the ending of the stroke—how the element is closed—in *hiragana* (Figure 1b): (1) “hooked” (brief stop followed by the abrupt reversal of pen tip movement at the end), (2) “sweep” (fast sweeping motion of the pen tip without change of direction), and (3) “stop” (slowing down to stop the pen tip to end the stroke). Every element in *hiragana* letters has either one of the above three types of ending, although there is variation in direction.

In Japan, prior to entering primary school, language education officially consists of verbal expression and listening (Ministry of Education, Culture, Sports, Science & Technology in Japan, 2011). It is not until the first months of the first grade when the official education of writing and reading begins. However, by the time the official education starts at the age of 6, most Japanese children have had some experience with writing implements and picture books, and have become able to write *hiragana* graphemes in their idiosyncratic manners more or less correctly in terms of their gross forms. The aim of the present study is to examine how such idiosyncratic patterns of movements involved in handwriting change when children are first exposed to a populated, classroom environment.

The main hypothesis of the present study was that *the social norms shared in a classroom environment influence the development of movement dynamics of handwriting*. Traditionally, cultural ecology of classroom learning and dynamics of handwriting movements have been studied and reported in different disciplines such as education and biomechanics (e.g., Graham et al., 2008; Shim et al., 2010). Nevertheless, to address the issue of how the former entrains the latter, the analyses of both ecological context and individual development are inevitably required. The originality of the paper lies in the attempt at finding connection between these different aspects of the development of children. To test the hypothesis that the development of movement dynamics of handwriting is canalized by what is valued in the classroom environment, the present paper analyses both (1) the ecological context of classroom setting and (2) the performance of emerging motor skills of children who participated in the classroom environment.

## 2 STUDY 1: DESCRIPTION OF A CLASSROOM ENVIRONMENT

The first step was to describe a classroom environment. Unlike other classroom studies involving a large number of samples (e.g., Graham et al., 2008), this section does not aim to present a representative picture of 1st‐grade language education. Rather, the aim of this section is limited to revealing the aspects of handwriting that are considered important in one classroom during the period of *hiragana* classroom education. Japanese written language education officially starts with the intensive learning of 46 *hiragana* graphemes at the beginning of primary school. Japanese Ministry of Education, Culture, Sports, Science and Technology (2008) provides a general curriculum guideline in compliance with the Basic Act on Education, the School Education Act. The guideline states that out of 306 class hours (1 class hour = 45 min) allocated to Japanese language education in the 1st year of primary school, approximately 100 class hours should be allocated to writing education (including the composition works), and that the 1st graders are expected to learn to be able “to read and write *hiragana* and *katakana* (Ministry of Education, Culture, Sports, Science & Technology in Japan, 2008).” All primary schools use one of the five official textbooks for Japanese language education. Although flexibility is given to schools and teachers to formulate curriculums, the variation in the design of classroom learning in Japanese primary schools is within a definite range reflecting the above requirements.

### 2.1 Method

At Kobe University Elementary School where the present study was conducted, *hiragana* lessons lasted for 10 weeks, three to four class hours per week (Table 1). During 10 weeks of *hiragana* education, typically, two letters are introduced and taught in each class hour. The materials used in this study include eight class hours of teaching 14 letters videotaped weekly from the 3rd to 9th week of *hiragana* education (2 class hours were recorded in the 3rd week, and one class hour in each of the following weeks). Lessons in the 1st and 2nd weeks were not videotaped for school reasons. The last day of *hiragana* education (10th week) was not reported because the lesson consisted of learning marks and symbols that go with *hiragana*, the emphasis of which were different from previous lessons. The parents of all the students gave informed consent prior to being videotaped and were not compensated for participation.

**Table 1:** Schedule of the first 12 weeks of Japanese education in 1st graders at Kobe University Elementary School and the timeline of the data analyzed for the present study

Week	Class contents	Data analyzed
1	*Hiragana* letters	—
2	*Hiragana* letters	—
3	*Hiragana* letters	Classroom observation
4	*Hiragana* letters	Classroom observation + Experiment 1st session
5	*Hiragana* letters	Classroom observation
6	*Hiragana* letters	Classroom observation + Experiment 2nd session
7	*Hiragana* letters	Classroom observation
8	*Hiragana* letters	Classroom observation + Experiment 3rd session
9	*Hiragana* letters	Classroom observation
10	Additional symbols	Experiment 4th session
11	(*Katakana* letters)	—
12	(*Katakana* letters)	Experiment 5th session

### 2.2 Results and discussion

#### 2.2.1 Characteristics of classroom activities

**Figure 2:**
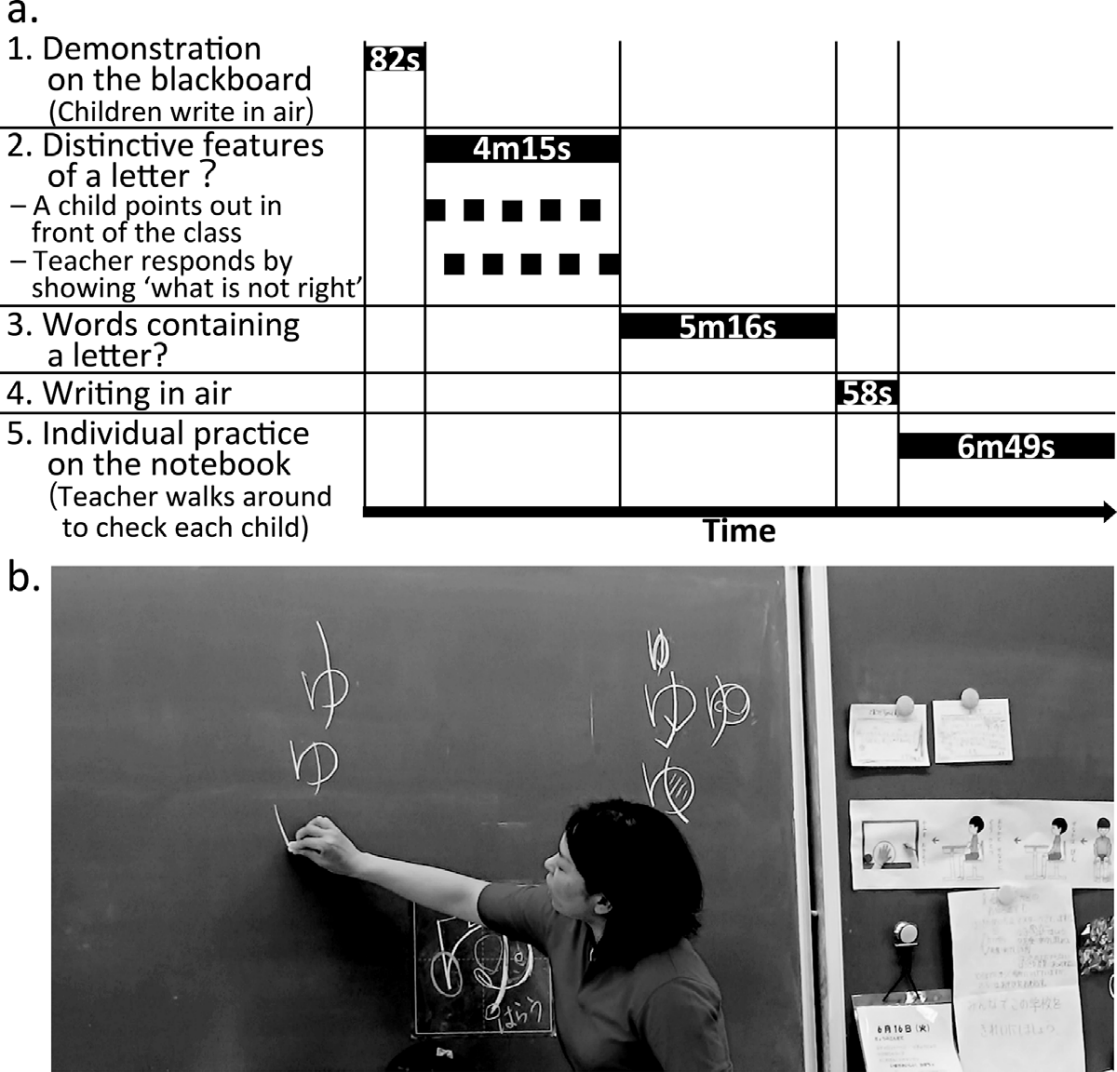
(a) Sequence of classroom activities in learning one *hiragana* letter (numbers indicate the average duration of each unit across lessons of 14 letters). The above sequence was usually repeated twice so that two *hiragana* letters were learned within one class hour (45 min). (b) Teacher responding to a child's comment by showing “what is not right.” Surrounding the model letter written below, “improper” variations of the letter had accumulated

In each class hour, a couple of new *hiragana* letters were introduced and taught. The sequence of classroom activities to teach a new letter remained fairly stable across the entire period of observation, which was repeated until all 46 letters were taught. For each letter, *hiragana* lessons moved through the following five classroom activities in sequence (Figure 2a): (1) teacher's writing demonstration on the blackboard; (2) public discussion on distinctive features of the letter; (3) children naming the words containing the letter; (4) children writing in air following the movement of the teacher; and (5) individual practice on the notebook.

During classroom practice, the aspects of the act of writing a letter frequently addressed by the teacher were classified into the following categories: (a) ending of the stroke (e.g., stop, sweep, or jump at the end); (b) curve/straightness of the stroke (e.g., make the stroke slightly curved); (c) turning of the stroke (e.g., make an acute angle); (d) intersection of the strokes (e.g., cross over the stroke); (e) proportions (e.g., make it shorter than the other element); (f) beginning (where to begin the stroke); (g) direction/slant of the stroke (the direction of the stroke or how it is to be slanted); (h) order of the strokes; and (i) rhythm (e.g., slowly here).

**Figure 3:**
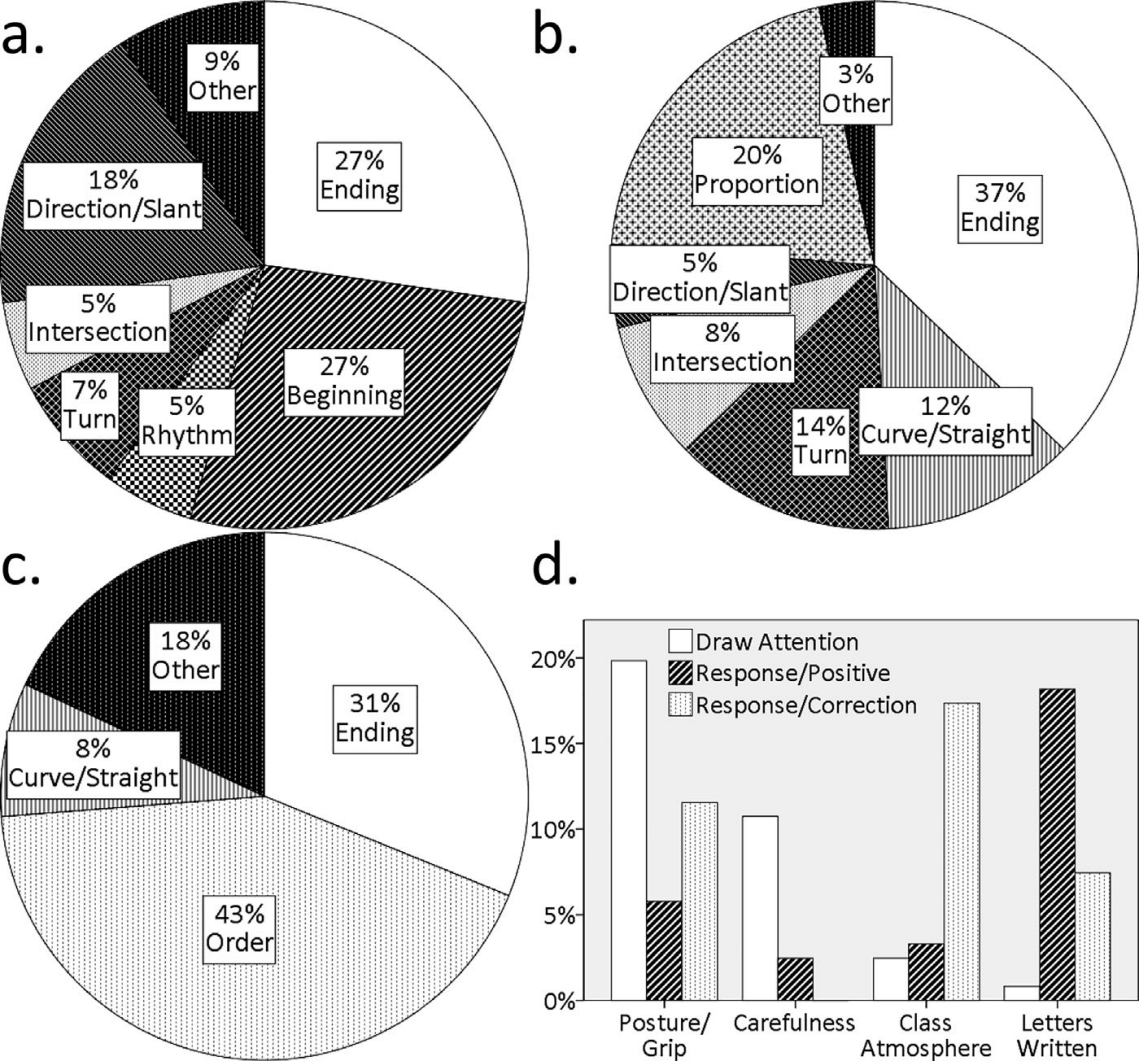
Proportion of the types of comments made by the teacher in the following segments of classroom practice: (a) demonstration on the blackboard, (b) distinctive features of the letter, (c) writing in air, and (d) individual practice on the notebook. Categories less than 5% are collapsed in the pie charts. This figure is based on the coding done by the author, which agreed well with the codings by the other two raters (intra‐class correlation coefficients across the three raters were always above .94)

The author and two other coders (Japanese graduate students majoring psychology) coded all the comments and responses made by the teacher independently for each classroom activity. The intra‐class correlation coefficients across the three raters were above .94 in all the segments of classroom practice coded, indicating high inter‐rater reliability.

In what follows, I will report on the characteristics of classroom interaction observed in each of the above classroom activities.

1. *Demonstration on the blackboard*: Without exception, lessons started with a teacher's demonstration of writing the letter‐to‐be‐learned on a square sheet attached on the blackboard. The sides of the square was about 30 cm long, and the square is ruled in such a way to divide the square into four small “chambers,” so as to make it easy for children to know the relative position of each element composing a letter. Children were encouraged to raise their hands and trace the strokes rhythmically in the air with broad gestures of arm and hand, closely following the teacher's writing movement on the blackboard. As the teacher writes on the blackboard, she would draw children's attention to the aspects of writing such as from which “chamber” one should begin the stroke, whether one should stop, sweep, or change direction of the pen movement at the end of the stroke, and so on. Out of 55 comments made by the teacher in this classroom activity over the entire observations (four comments per letter on average), the majority of the comments were about how to begin and end each stroke (Figure 3a).
2. *Distinctive features of the letter?*: After the demonstration on the blackboard, the teacher would ask children what one needs to pay attention to when writing the letter, lowering the position of the square sheet on the blackboard so that each child can come up in front and point at the part of the letter to be addressed (Figure 2b). When a child was picked by the teacher and commented in front of the class, the teacher was never observed to judge whether the comment is correct or not. Instead, the teacher typically responded by exaggerating the feature commented on by each child in a manner contrary to that suggested by the child on the blackboard. For example, when a child said “one should ‘sweep’ at the end of this stroke,” the teacher would respond by writing the same stroke with a “hooked” ending on the blackboard exaggerating how improper the letter would look with a stroke ending in an incorrect manner, saying, “so you mean, you are *not* supposed to write like this.” Such interaction was repeated with each children, and the “improper” variation of the letter in question accumulates on the blackboard, implying the variation of the letter that could not be tolerated (Figure 2b). Out of 59 teacher's comments in response to children in this classroom activity (five children were picked per letter on average), 37% was on the ending of the stroke and 20% was on the proportion among different elements of the letter (Figure 3b).
3. *Words containing the letter?*: Next, the teacher would ask children to name the word that contains the letter in question. No comments are made on the act of handwriting during this activity.
4. *Writing in air*: The teacher would say, “get your finger pencil ready,” encouraging children to raise their hands in the air. As the teacher traces the letter on the blackboard, children closely follow the teacher's movement rhythmically in the air with broad gestures of arm and hand. While executing the movement, the teacher would mention the aspects of writing one should pay attention to. For example, she would say, “one, briefly stop at the end, and jump to the other direction,” highlighting the order of stroke, and how one should end the stroke. Typically, this “writing in air” was done twice per each letter. Out of 85 comments made by the teacher during this activity, 43% was about the order of the strokes, 31% was about the ending of the strokes, and 8% was about the curve and straightness of the stroke (Figure 3c).
5. *Individual practice on the notebook*: Following the series of whole‐class activities above, children were encouraged to practice handwriting individually on the notebook. As children work on the notebook, the teacher would walk around the classroom and respond to each child by marking on their notebooks. In this classroom activity, the teacher's comments were quite different from those in the previous ones, which were classified into the following categories: (1) posture (including how to hold a pen and where to place the other hand); (2) carefulness in handwriting (e.g., write carefully and slowly); (3) letters written by children (e.g., you write very good); and (4) class atmosphere (e.g., be quiet). The type of comments on the above aspects were further classified as follows: (a) simply drawing attention (e.g., how are you supposed to sit when you write?); (b) positive comments in response to the activity of a child; and (c) corrections in response to the activity of a child (e.g., stop talking with your neighbors). Out of 122 comments made by the teacher during this activity, 20% were comments that drew children's attention to their postures, and 11% to the carefulness in writing (Figure 3d). A total of 17% were positive comments about the letters written on the notebook by children. Teacher corrected children's activity in response to the class atmosphere (17%, often telling them to be quiet), their postures (12%), and the letters they had written (7%). This activity was usually the final part of the lesson, whose mean duration was 6 min 49 s—the longest among the five classroom activities.

#### 2.2.2 Timeline of instructional interventions

**Figure 4:**
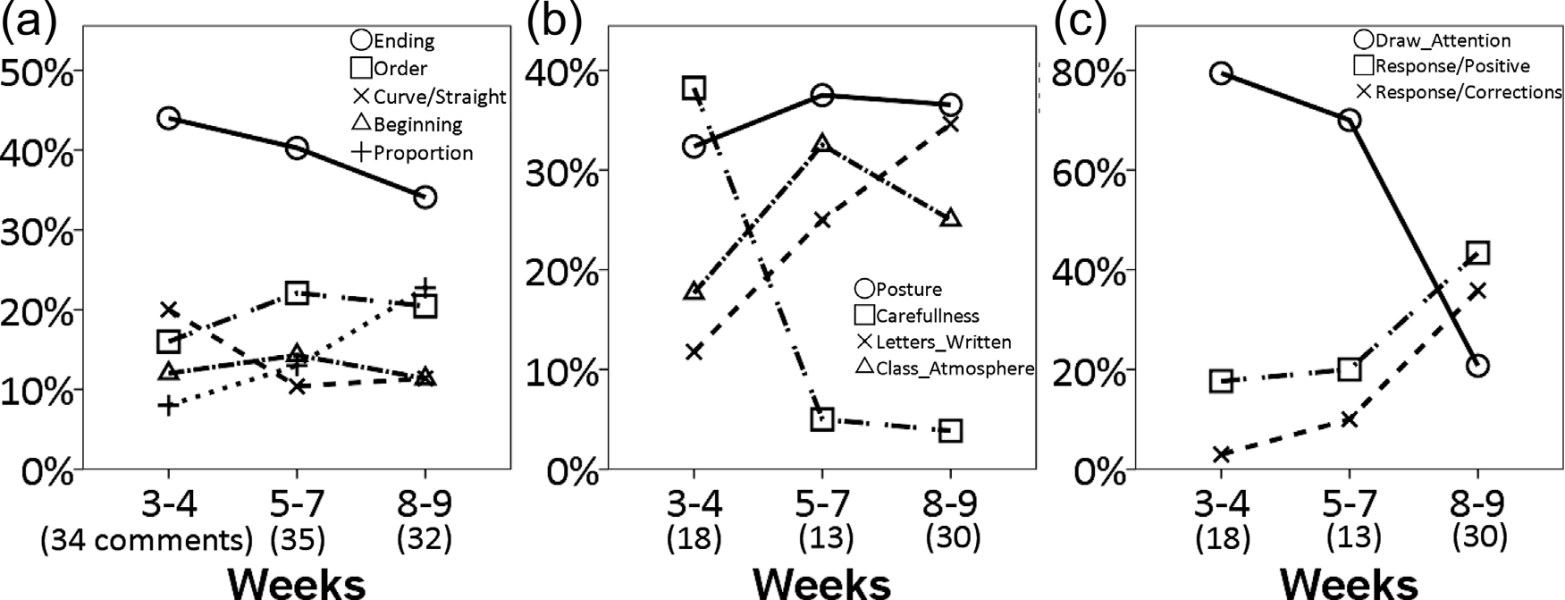
Changes in instructional comments provided by the teacher during (a) whole‐class group activity, and (b, c) during individual practice on the notebook over the period of observation. The numbers in parentheses below the axis labels of “Weeks” are mean frequencies of comments per class hour in the three periods

Despite the same sequence of classroom activities repeated for each letter, the instructional comments provided by the teacher exhibited both change and nonchange over the period (Figure 4). In the whole‐class activities, the teacher often highlighted the ending and the order of the strokes composing a letter throughout the period of *hiragana* learning (Figure 4a). During individual practice on the notebook, children were encouraged to write carefully in the early phase, while the teacher's instructional responses to the letters written by children increased over time (Figure 4b and c). It is also clear that posture was considered important when writing on the notebook (Figure 4b).

In summary, the observations of classroom leaning of 14 *hiragana* letters suggested several characteristics of teacher–children interaction that occurred in this classroom environment. First, interactions between the teacher and children were often mediated by the ongoing act of writing, which involved shared rhythmic motor experience between them. Second, the classroom interactions were structured in such a way for each child to see and hear the interactive responses of the teacher to how children have perceived and acted. Third, children were systematically encouraged to attend to certain aspects of the act of writing in the classroom environment, for example, movement with which one ends each stroke composing a letter. While some aspects of handwriting were highlighted throughout the period of *hiragana* classroom learning, there were also changes in the instructional comments provided by the teacher over the period.

## 3 STUDY 2: DEVELOPMENT OF HANDWRITING SKILLS

This part of the study aims to explore how the motor development of children using a stylus was modulated by the experience in this classroom environment. Since this is an exploratory study, I focused on general questions such as the following: Do the developmental changes of movement dynamics in handwriting of children reflect what was considered important in the classroom environment they were immersed, such as the differentiation of movements to end a stroke? Do the developmental changes of children accompany the increase in consistency not only of the form of a letter and a stroke but also of the rhythm of movement corresponding to each letter and stroke, whose experience was often shared in the classroom environment? Detailed data are provided about the different aspects of the characteristics of these changes. Data on the changes in movement dynamics of children during the classroom learning of non‐Latin alphabet *hiragana* have not been available in literature.

### 3.1 Method

#### 3.1.1 Participants

Participants were six 6‐year‐old 1st graders, three girls and three boy, with no known sensory or motor impairments. All participants attended the Kobe University Elementary School in 2015–16 school year. The three youngest girls and three youngest boys of the 35 children in the class were selected as participants, who were all born within the 2‐month period from February to March 2009. All children were right‐handed. The children were from Japanese families, who consented to participate in the experiment without compensation.

#### 3.1.2 Materials and apparatus

Children were instructed to write on the sheet of ruled paper attached on top of the digital tablet with active area 32.51 × 20.32 cm^2^ (Wacom Intuos 4, model PTK‐840, Wacom Technology Corporation; Vancouver, WA) with a digital inking ball point pen (Wacom Intuos Ink Pen, model KP‐130). MovAlyzeR V6.1 software (NeuroScript, LLC; Tempe, AZ) was used to record the position‐time data of the pen tip on the paper plane, pressure on the pen tip, and pen‐tilt relative to the paper plane from the digital tablet and the pen at 100 Hz. The software was also used to filter the recorded data. All trials were videotaped simultaneously with a digital video camera. The experiments were conducted in the same classroom as the one used for classroom learning of *hiragana*.

#### 3.1.3 Procedure

The trace‐making movements of the stylus manipulated by each child when writing *hiragana* letters were recorded every other week. The first session was conducted in the 4th week of classroom *hiragana* learning, and the final session was done in the 12th week (i.e., recording sessions are done in 4th, 6th, 8th, 10th, 12th week—five sessions in total). Note that *hiragana* education lasted only for 10 weeks, and the 12th week was two weeks after the completion of *hiragana* letter learning classes (Table 1). Except one boy who missed the first recording session, all the participants completed all the five recording sessions. Each child was presented a sheet of ruled paper and was instructed to write each of two to three Japanese words for five times between two vertical lines whose inter‐line distance was 1.4 cm. The model of the words to be written were printed on the right‐hand end of the same sheet of paper. The dimension of the model letter presented was approximately 0.8×0.8 cm. The set of words to be written in each session was selected in such a way to always contain the same four *hiragana* letters which consisted of ten strokes in total.

#### 3.1.4 Data analysis

**Figure 5:**
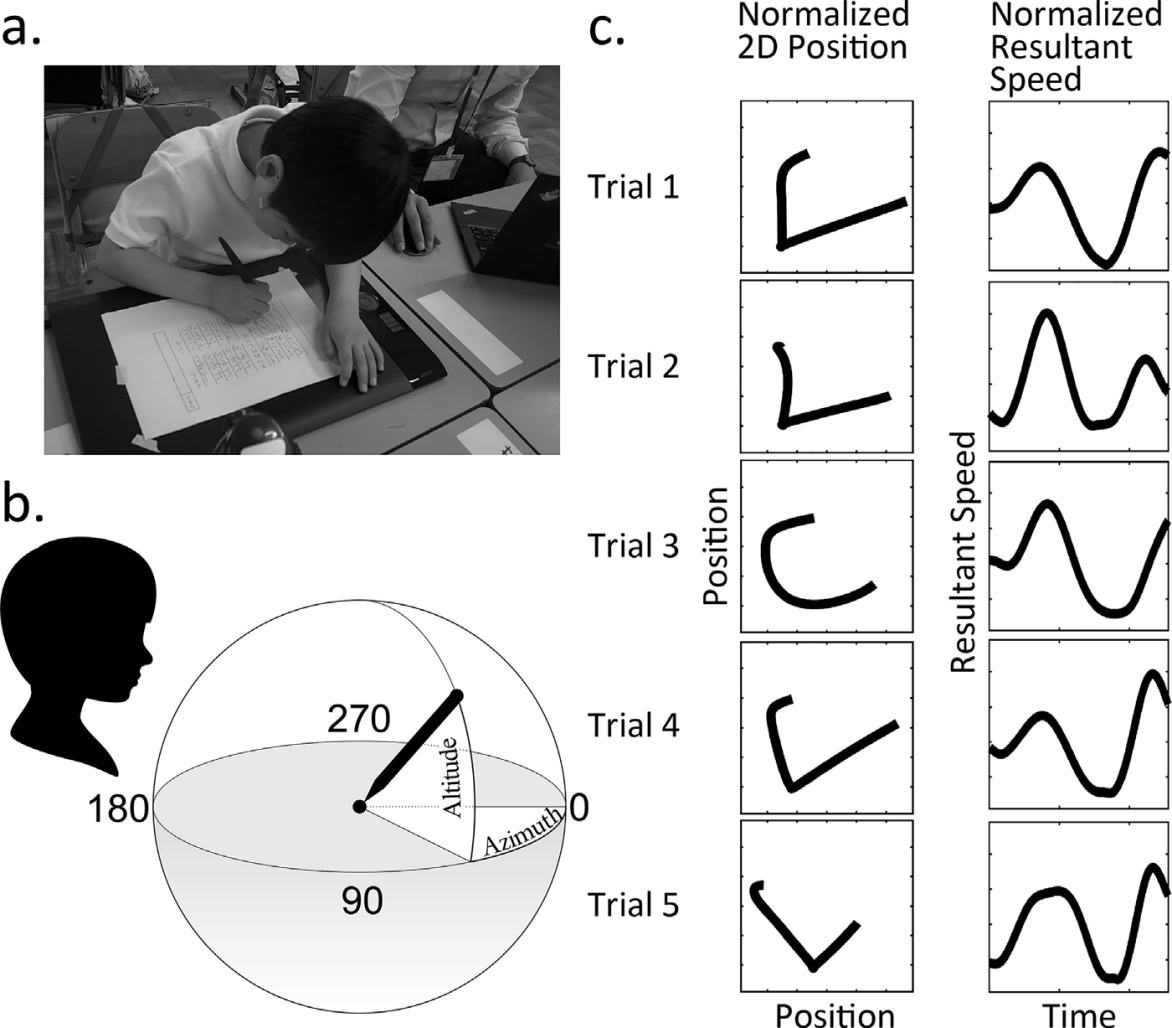
(a) Experimental set up. (b) Description of pen tilt in celestial coordinate system. (c) Example of normalized data of five trials of one subject from one session: Normalized two‐dimensional positions of a single pen stroke with “hooked” ending (left), and corresponding normalized resultant speed profiles of the pen tip from the same trials (right)

The horizontal and vertical position coordinates were low‐pass filtered at 12 Hz using a sinusoidal transition band from 7 to 17 Hz using multiplication of the spectrum (Teulings & Maarse, 1984). Filtered position coordinates were then segmented into 10 strokes consisting four *hiragana* letters in each trial. A very conservative approach was taken with regard to the measurements: if a part of a stroke consisting a letter written by a child fell outside the active area of the tablet, or if a stroke was interrupted and retouched afterwards by children, the whole stroke was excluded from the analysis. 1347 strokes in total contributed to the analysis (5 trials × 10 strokes × 6 children × 5 recording sessions, from which missing or excluded trials were subtracted). From the filtered horizontal and vertical position coordinates, trajectory length traveled by the pen tip, resultant speed of the pen tip on the paper plane, pen‐pressure (described as an integer within the range between 0 and 1024 pressure levels sensed by the digital inking ballpoint pen), and the orientation of a pen relative to a writing surface (described as azimuth and altitude in celestial coordinate system, see Figure 5b) of each stroke were computed. In addition, local maximum and minimum values of resultant speed time series (i.e., peaks) were picked using the minimum height difference between a peak and its neighbors of 1 cm/s with minimum separation of 100 ms between the peaks. These values were used to compute peak resultant speed of pen tip during each stroke, and to count the number of local minima that indexes the “wobbliness” of the pen tip movement (i.e., the greater the number of minima, the more wobbly the movement). The size (i.e., height and width) of each complete letter (consisting of multiple strokes), and the duration it took to write each complete letter were also computed (Table 2).

**Table 2:** Means and standard deviations of basic descriptive measures of *hiragana* handwriting across the six participants as a function of weeks after the start of *hiragana* class

Week	Duration (s)	Peak resultant speed (cm/s)	Vertical size (cm)	Horizontal size (cm)	Trajectory length (cm)	Pressure	Pen tilt azimuth (deg)	Pen tilt altitude (deg)	# of local speed minima
4	1.71 (3.89)	3.62 (1.59)	0.95 (0.30)	0.71 (0.23)	0.96 (0.59)	455.28 (193.33)	79.96 (22.59)	60.70 (6.82)	2.21(1.73)
6	1.92 (1.83)	3.75 (1.57)	0.96 (0.35)	0.73 (0.29)	0.93 (0.58)	412.72 (154.26)	88.10 (23.09)	58.09 (6.83)	1.94 (1.83)
8	2.11 (1.67)	3.85 (1.64)	0.98 (0.25)	0.73 (0.24)	1.01 (0.61)	397.71 (141.59)	88.79 (27.64)	57.21 (7.22)	1.55 (1.81)
10	1.89 (1.17)	4.76 (2.33)	1.23 (0.46)	0.88 (0.35)	1.19 (0.80)	437.26 (207.61)	96.56 (21.85)	58.23 (7.90)	1.34 (1.45)
12	1.74 (1.15)	4.62 (2.10)	1.12 (0.33)	0.76 (0.27)	1.08 (0.63)	459.92 (186.93)	93.95 (18.81)	60.18 (7.77)	1.31 (1.30)

Duration, vertical size, and horizontal size are computed for a letter (consisting of multiple strokes). Peak resultant speed, trajectory length, pressure, pen tilt, and the number of local minima of resultant speed were computed for a single stroke.

To capture the difference in the three types of the ending of the stroke in *hiragana* letters (Figure 1b), the average resultant speed of the pen tip movement was computed during 50 ms before the pen was lifted at the end of each stroke.

In addition to the original dataset, the normalized dataset was also created from the original dataset. First, each time series corresponding to each stroke was time‐normalized to 256 samples using a piecewise cubic spline interpolation to allow for alignment across trials. Time‐normalized data were used to visualize the rhythmic pattern of the pen tip movement during the writing of each stroke. Moreover, to achieve a common scale without influencing the distribution information within each time series, time‐normalized data were further normalized by subtracting the mean and dividing by the standard‐deviation. Using the normalized dataset thus created, the mutual information—a measure of the dependence between two variables (Cover & Thomas, 1991)—was computed to quantify the conformity of the resultant speeds of pen tip as well as the pen tip positions (i.e., forms) across trials within each subject each week (Figure 5c). Between all possible pairs of trials of each subject in each week (i.e., five trials would make 10 pairs), the mutual information was computed for time‐normalized resultant speed time‐series, as well as for normalized two‐dimensional positions. The mutual information between a pair of variables X and Y was estimated using the histogram approach, in which the probability density function of each variable is approximated using a histogram (Fraser & Swinney, 1986). Then, the mutual information (MI) between a pair of variables *X* and *Y* can be calculated according to the following equation (Cover & Thomas, 1991):

(1) $$MI\left(X;Y\right)=\overset{}{\underset{X,Y}{\sum }}P\left(X,Y\right){\text{log}}_{2}\frac{P\left(X,Y\right)}{P\left(X\right)P\left(Y\right)}.$$

For each histogram bin, the joint probability distribution P(X,Y) is estimated by counting the number of cases that fall into a particular bin and dividing that number with the total number of cases. The same technique is applied for the histogram approximation of the distributions P(X) and P(Y). The number of bins in the histogram was calculated by the Freedman–Diaconis rule using 10 randomly sampled actual data from the experiments, based on which the use of 10 bins was empirically selected (Freedman & Diaconis, 1981).

All measurements were calculated for each individual stroke for all participants (1347 strokes in total). A linear mixed‐model analysis was used to test the effect of “week” and/or “type of stroke ending” on the means of each selected variable. To account for the correlation between strokes made by the same participant, a participant factor was included as an additional random‐effect (Boyle & Willms, 2001). Bonferroni‐adjusted pairwise comparisons based on estimated marginal means were used for multiple comparisons between skill levels. All statistical tests were made using SPSS 22.0. The alpha value for a significant effect was set at 0.05.

### 3.2 Results and discussion

The modulation of handwriting movement during the period of official *hiragana* education were different across individuals, although there were also some common transformational invariants (c.f., Thelen et al., 1993). To capture this variability in developmental trajectory and to discover common strategies as well, the results section was divided into two sections: (1) collective results of all children and (2) examples of the developmental paths taken by individual children.

#### 3.2.1 Collective results

**Figure 6:**
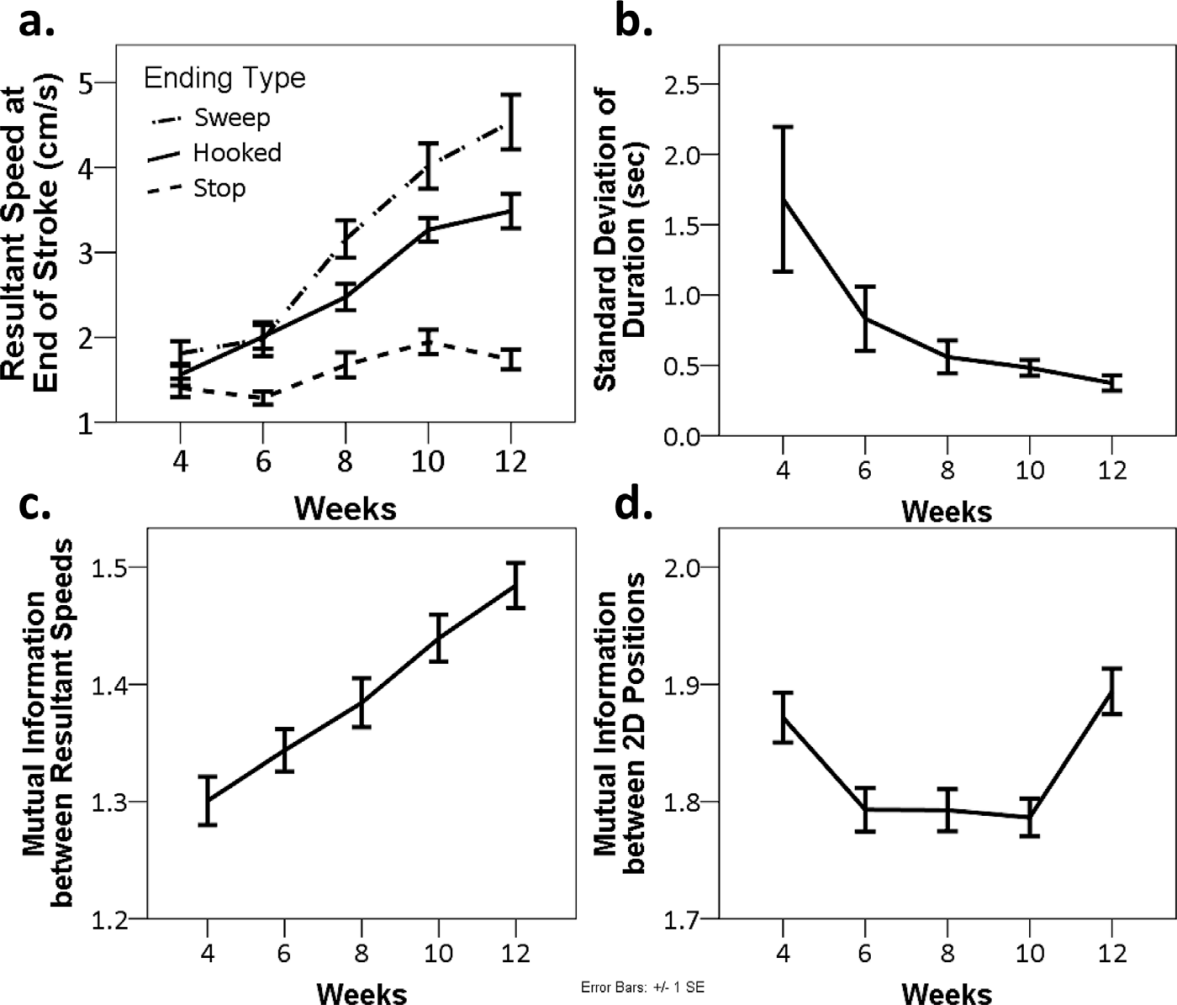
(a) Average resultant speed of pen tip movement within a 50 ms‐time window at the end of stroke as function of type of stroke ending and week. (b) Standard deviations of duration it took for children to write each *hiragana* letter as function of week. (c) Mean values of mutual information between all pairs of normalized resultant speed of stroke within each participant as function of week. (d) Mean values of mutual information between all pairs of normalized 2‐dimensional positions of stroke within each participant as function of week. Data from all children are pooled

In the previous section, we have seen that children were systematically encouraged to attend to the movement with which one ends each stroke composing a letter. As mentioned previously, in contrast to “stop” ending, “hooked” and “sweep” endings involve fast sweeping motion of the pen tip at the very end of the stroke. To examine the effects of classroom experience on the movements of children, the specificity of children's movements to these three different types of ending the stroke in *hiragana* was examined. A linear mixed model analysis on the resultant speed of the pen tip at the end of the strokes (average speed within a 50 ms window prior to lifting the pen) found a significant interaction between “week” and “type of stroke ending” [*F*(8,1325) = 8.69, *p *< .001]. The result showed that over the period of classroom learning of *hiragana*, the pen tip speeds at the end of strokes became gradually differentiated in such a way to specify the strokes with different types of ending (Figure 6a). Visual inspection of Figure 6a suggests that while the resultant speeds of the pen tip in the strokes with “stop” ending did not change over the weeks, those of the strokes with “hooked” and “sweep” endings increased dramatically. Bonferroni‐corrected post‐hoc analysis confirmed this impression. While there was no significant difference across the three types of ending at the 4th week of *hiragana* class, after the 6th week, resultant speed at the end of the strokes with “hooked” and “sweep” endings were consistently greater compared to the strokes with “stop” ending (*p *< .05) (Figure 6a). Accompanying such divergence specific to different ending types, the overall increase of peak resultant speed over the course of two months was also observed (Table 2).

While there was no consistent decrease of average duration it took for children to write one *hiragana* letter (Table 2), the variability of the duration across trials appear to have decreased over the period (Figure 6b). A linear mixed model analysis on standard deviations of duration found a significant main effect of “week” [*F*(4,281) = 4.51, *p *< .01], suggesting that the time it took for children to write each stroke became more consistent over the period of classroom *hiragana* education. The wobbliness of pen‐tip movement indicated by the number of local minima of resultant speed of pen tip also decreased (Table 2), which was shown to be statistically significant by the linear mixed model analysis on that measure which found a significant main effect of “week” [*F*(4,1336) = 5.36, *p *< .001].

I now turn to the issue of specificity of the rhythmic movement pattern *within* each subject to the written stroke. To test whether the temporal structure of the movement writing each stroke had become more consistent within each subject over the 2‐month period of *hiragana* education, a linear mixed model analysis on the mutual information between all pairs of time‐normalized resultant speed time series of the pen tip (i.e., 5 trials in a session yields 10 pairs of time series and 10 mutual information measures) was conducted. A significant main effect of “week” was found [*F*(4,2389) = 17.90, *p *< .001] (Figure 6c). Visual inspection of Figure 6c suggests that the mutual information, which reflects the consistency of the velocity profile of each stroke, increased over the period of *hiragana* classroom lessons. Bonferroni‐corrected post‐hoc analysis confirmed this impression by showing the mutual information was significantly greater in 10th and 12th week compared to the previous weeks (*p* < .05) (Figure 6c). This result suggests that each individual gradually assumed his or her consistent motoric pattern for writing each stroke during the period of classroom learning of *hiragana*. Note that what this result shows is the increased conformity of resultant speed profile of the tip of the pen *within* each subject, but not the conformity *across* different subjects. In other words, each individual exhibited the pattern of development in such a way to move the tip of the pen in a consistent manner over multiple sessions, yet the manner of movement may still differ across individuals.

Furthermore, to test whether the similarity of the overall form of the written stroke within each participant has increased, the linear mixed model analysis on the mutual information between all pairs of two dimensional pen tip positions was conducted. Although a significant main effect of “week” was found [*F*(4,2391) = 7.05, *p *< .001], the similarity of the written forms within a session did not increase linearly (Figure 6d). When the children gradually assumed the consistent rhythmic patterns for writing the strokes, there was no obvious corresponding change in the conformity of forms among the written strokes. The reason for this phenomenon is not entirely clear. One possible explanation is that when the rhythmic pattern of movement involved in writing a given stroke was undergoing transformation, the variability of the resulting traces increased momentarily. Or, this could be related to the nature of the analysis that took into account the conformity of normalized pen tip trajectories in two‐dimensional coordinates without considering those invariants that make these trajectories *hiragana* letters.

The way in which children oriented a pen to a writing surface changed only a little. The children, who were all right handed, tilted the pen to their right‐hand side from the beginning, the tendency of which seems to have gotten slightly more pronounced in the later sessions compared to the earlier sessions (Table 2). Apparently, there were no common, consistent changes across children in the size of the letters written, the total length of the pen tip trajectory of one stroke, the pressure at the pen tip, and the altitude of the pen in celestial coordinate system (Table 2).

#### 3.2.2 Examples of the paths of individual development

In what follows, detailed data on two girls who exhibited quite different developmental trajectories were selected and presented with the aim of highlighting the paths taken by different individuals underlying the collective results reported above.

##### The case of Yui

###### First recording

**Figure 7:**
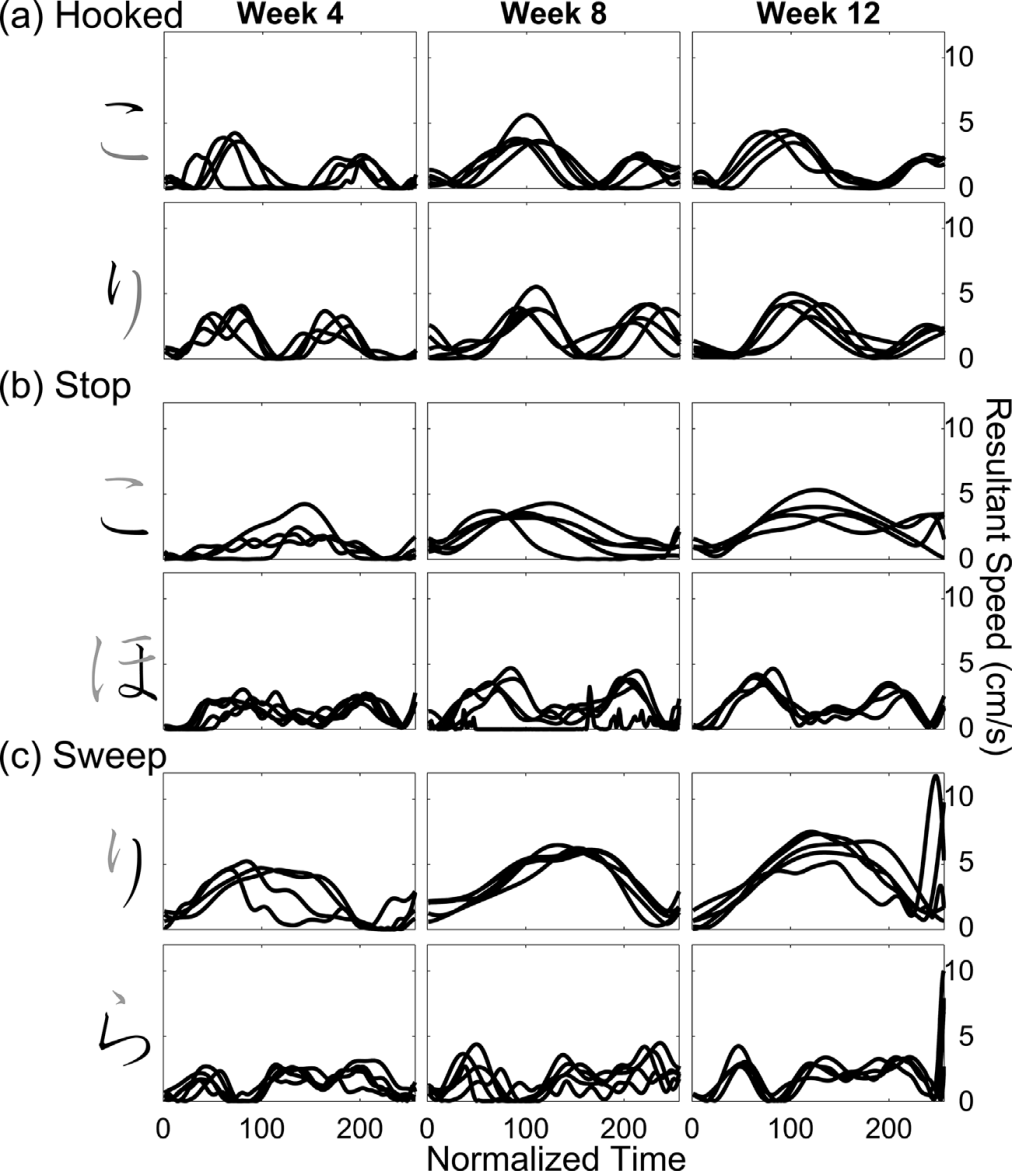
Time‐normalized resultant speed profiles of pen‐tip when Yui wrote the stroke with (a) “hooked” ending, (b) “stop” ending, and (c) “sweep” ending, at 4, 8, and 12 weeks after the beginning of *hiragana* class. The black element of a *hiragana* letter on the left correspond to the movement profiles shown on the right. Overlaid lines show multiple trials recorded at each week

**Figure 8:**
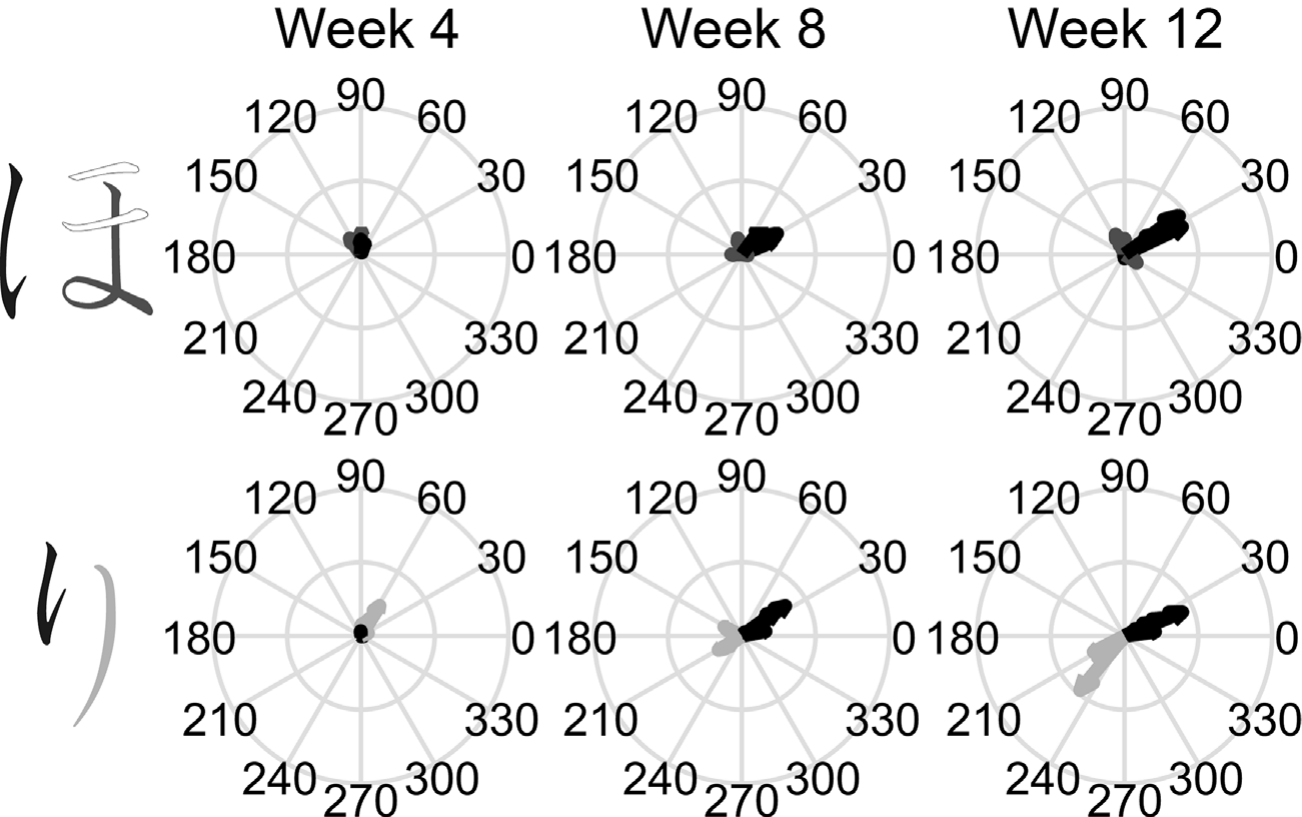
Progressive differentiation of the pen‐tip movement of Yui between strokes with different endings composing *hiragana* letters. Black, dark‐gray, and light‐gray arrows on the right correspond to the ending velocities of pen‐tip writing the elements with “hooked,” “stop,” and “sweep” endings of *hiragana* letter shown in black, dark‐gray, and light‐gray on the left, respectively. The size and direction of arrows show the magnitude and direction of average velocity within a 50 ms‐window prior to lifting the pen. Overlaid arrows with the same color correspond to the multiple trials recorded at each week

The kinematic qualities of Yui's pen tip movements when she was writing strokes with different types of ending are illustrated in Figure 7. At the end of the stroke, the resultant speed was at the “valley” close to zero irrespective of the type of stroke ending in the first session (left panels in Figure 7). Close inspection of Figure 7a further suggests that the strokes with “hooked” ending typically had two peaks in the 4th week, implying that the valley in the middle of resultant speed profiles in Figure 7a correspond to the moment where the reversal of direction of the pen tip in “hooked” ending occurred. After this change of direction, the pen tip would again accelerate and decelerate, and the ending of the stroke almost always corresponds to the valley after the second peak (Figure 7a). The tendency to slow down the movement at the end of all the strokes is also apparent in Figure 8, which presents the average velocity within the 50 ms‐time window at the end of each stroke as an arrow, whose size and direction correspond to the magnitude and direction of the velocity vector. In the 4th week, Yui almost always stopped the pen tip movement at the end of the stroke regardless of ending types (Figure 8).

###### Subsequent modulation

**Figure 9:**
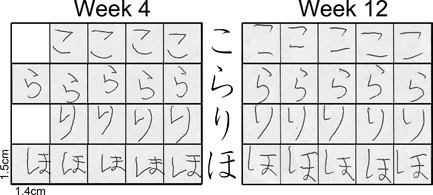
The four *hiragana* letters written by Yui in the first (left: 4 weeks after the start of *hiragana* education) and last recording sessions (right: 12 weeks after the start of *hiragana* education)

**Figure 10:**
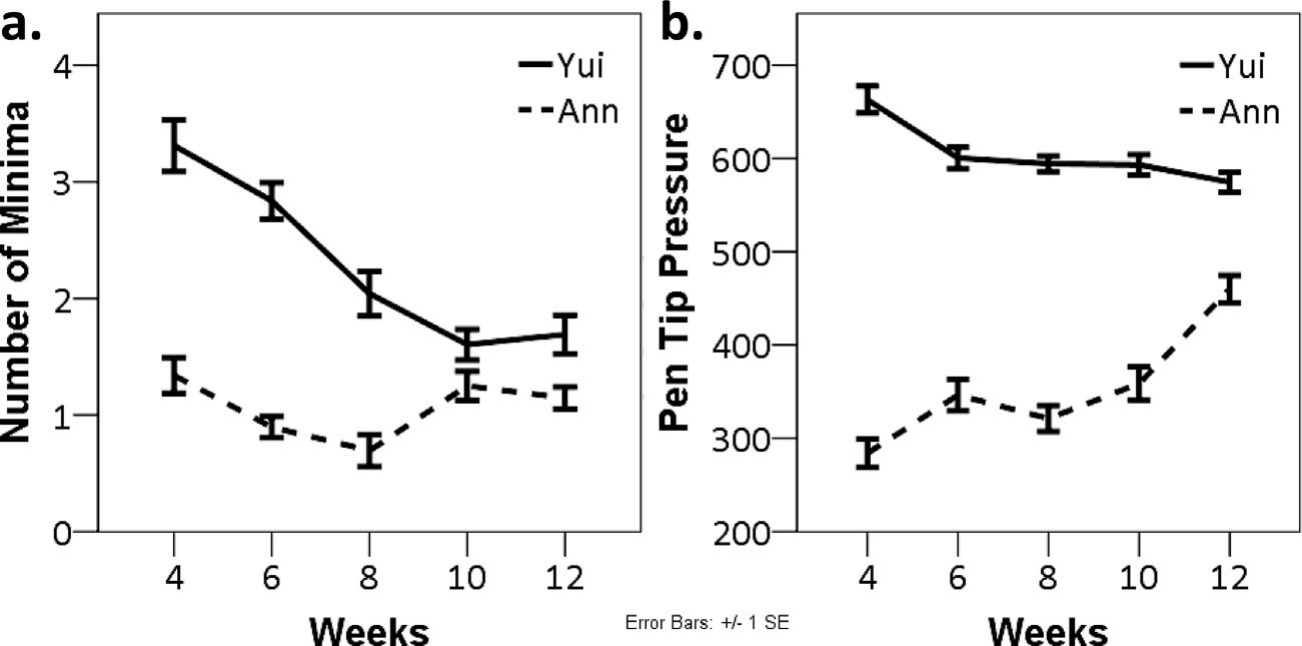
Mean values for the following measures for Yui and Ann by week: (a) number of velocity minima during a single stroke, and (b) pen tip pressure

Over the period of classroom learning of *hiragana*, Yui's movement gradually became differentiated across the strokes with different types of endings (Figure 8). In particular, the velocity at the end of strokes with “hooked” ending (black arrows in Figure 8) changed dramatically: The velocity increased, and their direction became more consistent across trials, especially after the 8th week. Similar tendency was also found in “sweep” ending (light‐gray arrow in the bottom panel of Figure 8). The resultant speed profiles also changed. The second peak of the strokes with “hooked” ending became progressively shifted backwards in such a way to end at the peak of speed instead of its valley after the peak (Figure 7a). Overall, visual inspection of Figure 7 suggests that by the 12th week, the movement of the Yui had assumed consistent rhythmic patterns specific to the type of strokes. Reflecting the increased pen‐tip speed at “hooked” ending, the written traces at the end of “hooked” strokes were thinner in the 12th week compared to the 4th week, and the forms of letters differed across these sessions in details of connections between strokes (Figure 9). The wobbliness of Yui's pen tip movement that were initially present gradually disappeared (Figure 7b,c), which was also shown by the decrease in the number of resultant speed minima in the later sessions compared to the earlier sessions (Figure 10a).

##### The case of Ann

###### First recording

**Figure 11:**
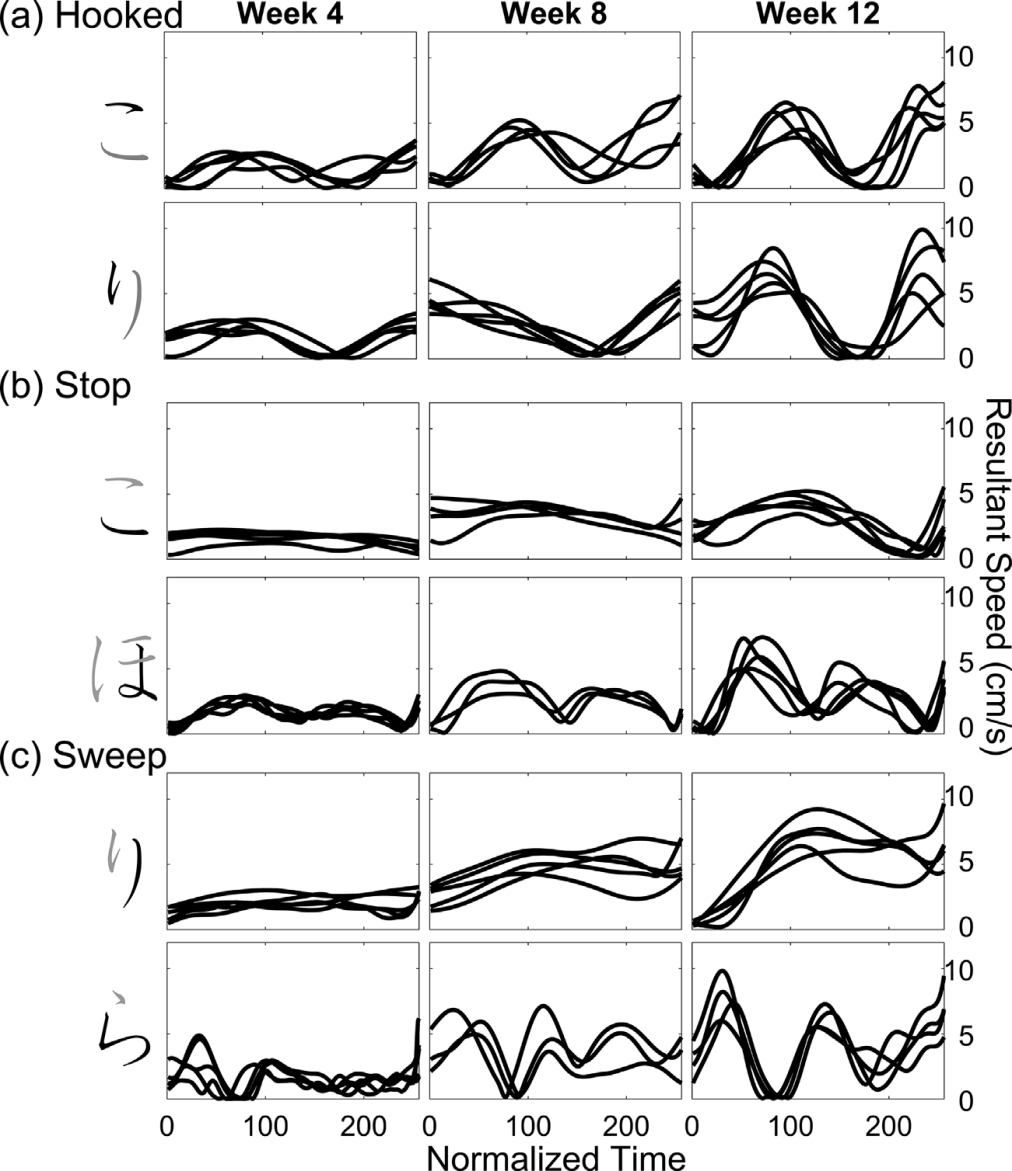
Time‐normalized resultant speed profiles of pen‐tip when Ann wrote the stroke with (a) “hooked” ending, (b) “stop” ending, and (c) “sweep” ending, at 4, 8, and 12 weeks after the beginning of *hiragana* class. The black element of a *hiragana* letter on the left correspond to the movement profiles shown on the right. Overlaid lines show multiple trials recorded at each week

The letters Ann wrote were very small in the beginning (Figure 13), her movements were relatively slow (Figure 11), and the pressure of the pen tip was also very low (Figure 10b). In contrast to Yui whose pen tip movement always reached a halt at the end of the strokes in the earlier sessions, Ann's relatively small and slow movements already exhibited the rhythmic patterns specific to different types of strokes from the 4th week, such as a brief stop followed by the abrupt jump at the end in “hooked” endings (Figure 11a). Her movements were a little wobbly when writing a winding stroke (the second row in Figure 11b,c), but overall, Ann exhibited mature‐looking patterns of movements compared to the other children from the beginning.

###### Subsequent modulation

**Figure 12:**
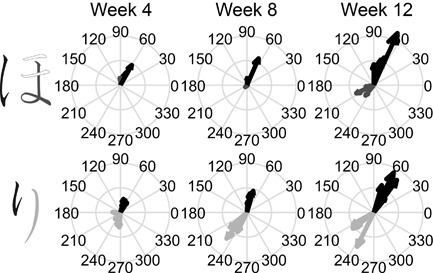
Progressive differentiation of the pen‐tip movement of Ann between strokes with different endings composing *hiragana* letters. Black, dark‐gray, and light‐gray arrows on the right correspond to the ending velocities of pen‐tip writing the elements with “hooked,” “stop,” and “sweep” endings of *hiragana* letter shown in black, dark‐gray, and light‐gray on the left, respectively. The size and direction of arrows show the magnitude and direction of average velocity within a 50 ms‐window prior to lifting the pen. Overlaid arrows with the same color correspond to the multiple trials recorded at each week

**Figure 13:**
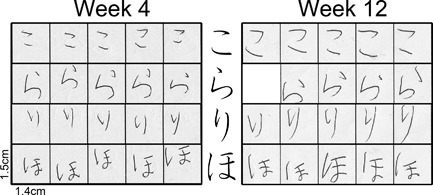
The four *hiragana* letters written by Ann in the first (left: 4 weeks after the start of *hiragana* education) and last recording sessions (right: 12 weeks after the start of *hiragana* education)

Ann scaled up her pen tip velocity in the subsequent weeks (Figure 11). Not only did Ann learn to produce higher pen tip velocity, Ann's movements were modulated in such a way to assume the pronounced specificity to the strokes with different types of ending (Figure 12). Note the difference in the magnitude of velocity between “hooked” stroke (black arrows) and “stop” stroke (dark‐gray arrows) in the upper panel in Figure 12. While Ann's pen tip movement reached an almost complete halt at the end of “stop” strokes (Figure 11b), her pen tip often moved with the utmost rapidity at the end of “hooked” and “sweep” strokes (Figure 11a,c). Another notable feature of Ann's writing in the final week is the remarkably consistent temporal structures of the pen tip movements, in which peaks and valleys of resultant speed were topologically similar across trials. At a first blush, the gross forms of letters written on the sheet in the final session seem as variable as the first session. But the subtle details such as thickness of lines at the end of each stroke (bottom or right end of each element) appear to be more differentiated across different types of strokes in the final session (Figure 13), reflecting the underlying dynamics of the trace‐making movements that had changed during classroom learning of *hiragana*. In other words, developmental changes did not appear uniformly in all aspects of letters written by Ann, but appeared selectively in subtle details.

Another interesting aspect of Ann's development was the control of the pressure at the pen tip. In contrast to Yui who exhibited the tendency to relax the pen tip pressure over sessions, Ann progressively scaled up her pen tip pressure against the paper (Figure 10b). Such difference suggests that the challenges faced by Ann in learning skilled handwriting behavior in the classroom environment might have been different from those faced by Yui. In sum, in the process of scaling up the velocity, pressure, and the magnitude of pen tip movements, what emerged in Ann's writing during the period of classroom learning were both (1) the clear differentiation of pen tip movements across different types of strokes and (2) the “throbs” of pen‐tip movement specific to the different strokes composing *hiragana*.

## 4 GENERAL DISCUSSION

This study reports, for the first time, detailed description of the process of how dynamics of fine motor movement is entrained by social training in a non‐Latin alphabet writing system. By the time the official education starts at the age of 6, most Japanese children have had some experience with writing implements and picture books, and have become able to write *hiragana* in their own ways. The aim of the present study was to examine how such idiosyncratic patterns of movements involved in handwriting change when children are first immersed in a populated, classroom environment where children are systematically encouraged to attend to certain aspects of the act of writing letters. To look into this issue, first, I focused on the structure of classroom events and the specific types of interaction and learning within such environment. Second, in the experiment involving six children who attended the class, I systematically analyzed the dynamics of movements underlying each stroke composing letters written by children, and their modulation during the whole period of *hiragana* education. Summarily, the results are as follows:

- The patterns of movements in children writing *hiragana* letters became differentiated in a manner specific to the different types of stroke endings over the first months of primary school.
- Increased conformity of the temporal patterns of pen‐tip movements over the period of classroom learning was observed within each individual, in such a way for a given letter to be written with a like temporal structure of movement.
- The developmental paths taken to differentiate movement patterns specific to the distinctive features of *hiragana* strokes were different across individual children.
- In the classroom, interactions between the teacher and children were often mediated by the ongoing “act” of writing, which involved shared rhythmic motor experience between them.
- The lessons in the classroom were structured in such a way for children to see the interactive responses of the teacher to how they have acted, in which a consensus had grown as to which aspects of the act of writing matter.

### 4.1 Transformations of pre‐existing patterns of handwriting movements

One of the basic yet surprising outcomes of the present study was that even within the short period of learning to write in a classroom environment, the patterns of handwriting movement in children underwent progressive transformations. At the time of first recording, the six children were already able to produce legible handwriting, but still, the continual exposure to the classroom environment somehow led to the remodeling of their pre‐existent patterns of handwriting movements in the subsequent weeks. What underlies such transformations of movement patterns? The results of the present study suggest that although the developmental paths taken were different across individual children, such transformations were not randomly directed, but there were some common transformational invariants in the directions toward which movement patterns have evolved.

On the 4th week of their first year in primary school, these children showed very different patterns of handwriting movements: Yui's movement reached a halt on a writing surface at the end of all letter strokes. Ann's handwriting movement was slow, small, and weak. Yet, as exemplified in Figures 8 and 12, both of them changed in such a way to differentiate the patterns of movement at the end of strokes with different ending types, despite the fact that somewhat similar form could still be produced with their previous patterns of movements (Figures 9 and 13). What are the reasons for such a differentiation? Study 1 showed that the movements with which one ends strokes composing a *hiragana* letter was consistently paid attention to in the classroom environment. The progressive differentiation of this aspect of handwriting movement during the first months of primary school is likely to be the result of learning to attend to these meaningful features and of becoming more sensitive to them in a populated environment. Recently, Maldarelli and co‐workers illustrated the development of visual attention of 5‐year olds during letter copying task (Maldarelli, Kahrs, Hunt, & Lockman, 2015). The extent to which the visual attention develops in conjunction with education of attention in the classroom environment during such tasks may be an interesting topic of future research.

Another question of interest is how children come to be sensitive to such subtle distinctive features of elements composing *hiragana*. The classroom observation showed the characteristic manner in which the teacher directed children's attention to aspects of writing such as the order of the stroke and how the stroke should be closed. In the case of the skills such as reaching or pointing, how children move the hand through space to contact a target, and the consequences of their self‐produced activity can be perceived with eyes, heads, mouths, and limbs through receptors in muscles, joints, skin, and vestibular system (Thelen et al., 1993). Thereby, a movement repertoire of a child can be transformed into adaptive actions through the continual process of exploration of the perceptual consequences of self‐generated movement (Sporns & Edelman, 1993). But, where is the information that specify the *social* consequence of self‐produced activity in relation to the social norms of a classroom environment to which children need to adapt? The classroom observation suggests that the classroom learning environment was structured in such a way for each child to immediately see and hear the interactive responses of the teacher as a consequence of the child's own perceived activity. In a public classroom activity, for example, a child pointed out what she considered critical in writing a letter, and the teacher responded by exaggerating the features in a manner contrary to that suggested by the child. When children practiced individually on their notebooks, teacher responded to what kind of posture each child assumed, and how they wrote on the notebook. The reciprocal activities of the teacher and children were co‐ordinated, and the teacher responded to children immediately and coherently. These responses, in relation to a child's own perceived activity, might serve to inform the child about his or her self‐produced activity in relation to what is considered important in a given populated environment (c.f., Neisser, 1988).

The data on the details of what transpires in the home environment during the period when *hiragana* is being taught at school was not collected in the present study. Although writing experience at home could vary across children, the teacher of the 1st grade class and the principal of the primary school where the present study was conducted pointed out the two kinds of experience that those children in the class have in common at home, which complements the instruction and experience that is given in schools. First, children are encouraged to write their names in *hiragana* at the beginning of classroom learning and to practice at home. Second, homework is assigned to children so that they can practice the letters that have been learned in the day's class. Both of these are usually conducted under parental supervision. The extent to which different experience in the home environment would affect how the development of letter writing unfolds may be another potential topic for future research.

### 4.2 Increased specificity in the temporal structure of the pen tip movement

The results of the present study suggest that what is learned by children includes the dynamic patterns of stroke when writing *hiragana*. Figures 7 and 11 clearly illustrate that the rhythmic patterns of pen tip movements of children—how the pen tip lingers, leaps, and stops on a writing surface—have evolved in such a way to assume a consistent, stroke‐specific temporal structure. In the collective data from all the six children, such specificity was exemplified by the increase of the mutual information between normalized resultant speeds between the same strokes within each child over multiple sessions (Figure 6c).

In the Japanese classroom where observations were made, learning of the letters were not cut off from their gestural foundations. The form of each letter was regarded as a result of dynamic movement, such as how you end each stroke and its transitions to the following stroke. In some of the classroom activities, children first execute the gesture in the air with rhythmical movements which were coupled to that of the teacher writing on the blackboard, carefully making the endings. Each child was encouraged to attempt at moving one's own body in a particular temporal sequence, which may help the child resonate to the critical features that distinguish one letter from another. Such emphasis on gesture may be characteristic of societies under the influence of Chinese writing system (Billeter, 1990). Sasaki's (1987) series of experiments convincingly showed that when Japanese people cannot recall a Chinese character, they search with the hand until the gesture performs itself and restores the forgotten form, just as we would to recall a forgotten dance step. In fact, they are so accustomed to “mock writing” that when there is any uncertainty about a word when writing, they trace the character in the air or on their palm, and this significantly contributes to dispelling the uncertainty (Sasaki, 1987). *Hiragana* has its origin in Chinese character, and has characteristics in common in the set of strokes composing letters. It is not improbable that the process of learning letters that involves rhythmic motorial experience is related to this specific word recall strategies seen in Japanese people.

Traditionally, it has been assumed that underlying the skill of handwriting is the stored effector‐independent visual representation of the strokes in handwriting (Wing, 2000), in which the two following tasks were said to exist: (1) the formation of the trajectory of the writing implement and (2) the production of the desired trajectory of the tip of the pen on a piece of paper (Latash, Danion, Scholz, Zatsiorsky, & Schöner, 2003; van Gemmert & Contreras‐Vidal, 2015). However, the results of the present study—that Japanese children acquire the specific temporal pattern of gesture corresponding to a stroke in the beginning of *hiragana* education—seem to suggest an alternative explanation. At least in this particular culture, what is learned may not be a picture of a letter plus the ability to trace that picture. Instead, it appears that learning of the temporal pattern of movement corresponding to a letter is primary, based on which the invariant features of a letter—the traces of the specific temporal pattern of movement—come to be discriminated as such. To ascertain meaningful invariants in the skill of handwriting across cultural variations, it would seem desirable that more studies on the development of handwriting in societies using non‐Latin alphabet writing systems be conducted.

On the other hand, the increased specificity of temporal structure of the pen tip movement does not necessarily imply that what is acquired by the children were a set of muscle activation patterns. Just as the same melody could be sung or whistled, the same temporal structure of the movement on the pen tip on a horizontal piece of paper can emerge from a number of different coordination patterns among fingers that make contact with the pen (Latash et al., 2003; Shim et al., 2010). Nonaka (2013) reported on the motor coordination of a tetraplegic calligrapher who write with a writing implement gripped between his teeth. The study illustrated the movement variability of the head and that of the cervical spine were coupled in a compensatory manner to stabilize the upright posture of the brush relative to the contact with the paper, as the head flexed down and up, and rotated, to produce the same strokes in multiple trials (Nonaka, 2013). In the present study, although the data collected were restricted to the movement of the pen tip and the pen's orientation relative to a horizontal surface, the progressive changes in the movement of the pen tip found in children that accompanied only slight changes in pen orientation may be indicative of motor equivalent control of the pen tip. Underlying change in the strategies to control a redundant system to produce a specific pen tip movement may be a topic of future research.

## 5 CONCLUSIONS

Traditionally, the development of handwriting has been studied as the development of a task of producing a desired trajectory of the tip of the pen on a horizontal surface. The present study extends the prior works by describing the development of handwriting of children in relation to the aspects of writing that are considered important in a particular social environment. The data allowed for the examination of how children individually found solution to match the opportunities and constraints of the body‐plus‐tool system to the social constraints and social norms of a classroom environment. The main finding of the present study is that handwriting movements of the six Japanese children underwent progressive transformations in such a way to differentiate a set of subtle features of elements of letters in the first three months of primary school. The children showed, by doing, the depth with which human cultural entrainment penetrates a case of “fine motor skill” development—the acquisition of handwriting skills.

## Supporting information

Additional Supporting Information may be found online in the supporting information tab for this article.

Supporting Video S1.

Supporting Video S2.

Supporting Video S1.

Supporting Video S2.
